# Research Hotspots and Frontiers of Ischemic Stroke and Traditional Chinese Medicine: A Knowledge Mapping and Text Mining Analysis

**DOI:** 10.2174/011570159X378811250702095624

**Published:** 2025-07-18

**Authors:** Jian Xie, Teng Li, Yuan Zhang

**Affiliations:** 1 Department of Neurology in Affiliated ZhongDa Hospital and Jiangsu Provincial Medical Key Discipline, School of Medicine, Institution of Neuropsychiatry, Key Laboratory of Developmental Genes and Human Disease, Southeast University, Nanjing, Jiangsu, 210009, China;; 2 Department of Laboratory Medicine, Shanghai East Hospital, School of Medicine, Tongji University, Shanghai, 200120, China;; 3 Department of Pharmacy, Shanghai East Hospital, School of Medicine, Tongji University, Shanghai, 200120, China

**Keywords:** Ischemic stroke, TCM, bibliometrics, neural regeneration, network pharmacology

## Abstract

**Background and Objective:**

Ischemic stroke is a complex neurological disease with poor prognosis, resulting in significant economic burdens for patients, their families, and society at large. Traditional Chinese Medicine (TCM) offers unique advantages and has been utilized for thousands of years in the prevention and treatment of ischemic stroke. However, there is currently a lack of bibliometric analysis and knowledge mapping regarding TCM treatments for this condition. To identify the key areas and future directions of TCM treatment for Ischemic Stroke.

**Methods:**

We extracted articles on TCM treatments for ischemic stroke from the Web of Science Core Collection (WoSCC) database, spanning from January 1, 1990, to March 20, 2025, and conducted a bibliometric analysis. Data processing was performed using CiteSpace, VOSviewer, and Origin2024b.

**Results:**

We collected 506 published papers, with China accounting for the majority of these publications (97.0%, 418 articles). The Journal of Ethnopharmacology and Frontiers in Pharmacology were the two most frequently published journals. The leading institutions contributing to this research included the Chinese Academy of Medical Sciences, Beijing University of Chinese Medicine, and Peking Union Medical College (now known as Capital Medical University). The primary research hotspots in TCM treatment for ischemic stroke focused on protective effects, brain injury, and neural regeneration. Notably, network pharmacology, molecular docking, and metabolomics were identified as the commonly used research methods. Among them, network pharmacology was the most frequently used research method. Additionally, we summarized the TCM remedies that have been extensively researched and cited in the context of ischemic stroke, including catalpol, angong niuhuang wan, buyang huanwu decoction, jeo-dang-tang, yiqifumai powder injection, and gualou guizhi decoction.

**Conclusion:**

The work highlights the key topics and recent research frontiers, providing valuable resources for scholars interested in the role of TCM in treating ischemic stroke.

## 1 INTRODUCTION

Ischemic stroke is a common neurological disorder associated with high mortality and severe disability rates worldwide [1, 2]. In the early stage of onset, thrombolysis and endovascular thrombectomy are the primary treatments for ischemic stroke [3]. However, due to time constraints, these therapies may not be effective for a large number of patients [4]. In this context, the development of multicomponent pharmaceutical preparations, such as traditional Chinese medicine (TCM), has high clinical value and social significance.

TCM has been utilized in clinical applications for over 5,000 years in China, where it is supported by a robust theoretical foundation and a wealth of clinical experience demonstrating its effectiveness [5, 6]. The complex cascading reaction process after stroke may be one of the reasons for the failure of clinical trials of multiple neuroprotective chemical agents in recent years [7-9]. Based on the benefits of multicomponent, multitarget, and multi-pathway effects, TCM has garnered worldwide attention and become one of the most essential resources for pharmaceutical and clinical medicine in identifying new drug candidates for treating diseases [10, 11]. In recent decades, TCM has gradually become a new research focus for improving functional recovery after ischemic stroke [12, 13]. However, there are currently no articles published that offer a systematic bibliometric analysis of TCM in ischemic strokes. Therefore, it remains a challenging task to comprehensively learn about the current research landscape of TCM in ischemic stroke.

Bibliometrics is one of the most common techniques for quantitatively analyzing and evaluating publications about a specific subject [14, 15]. It collected information on various entities (*e.g*., journals, organizations, and countries) to identify underlying correlations and facilitate a broader understanding of the research field [16, 17]. Bibliometrics, when combined with visualization tools, helps academics generate new study ideas and gain a comprehensive understanding of a rapidly evolving topic. This study analyzed applied research topics, hotspots, and novel perspectives of TCM in ischemic stroke using CiteSpace and VOSviewer. This work aims to summarize the major research themes, discuss the potential research directions, provide the main TCM for treating ischemic strokes, and investigate future directions for basic and clinical research of TCM treating ischemic stroke.

## 2 METHODS

### 2.1 Data Collection

**Fig. (1):**
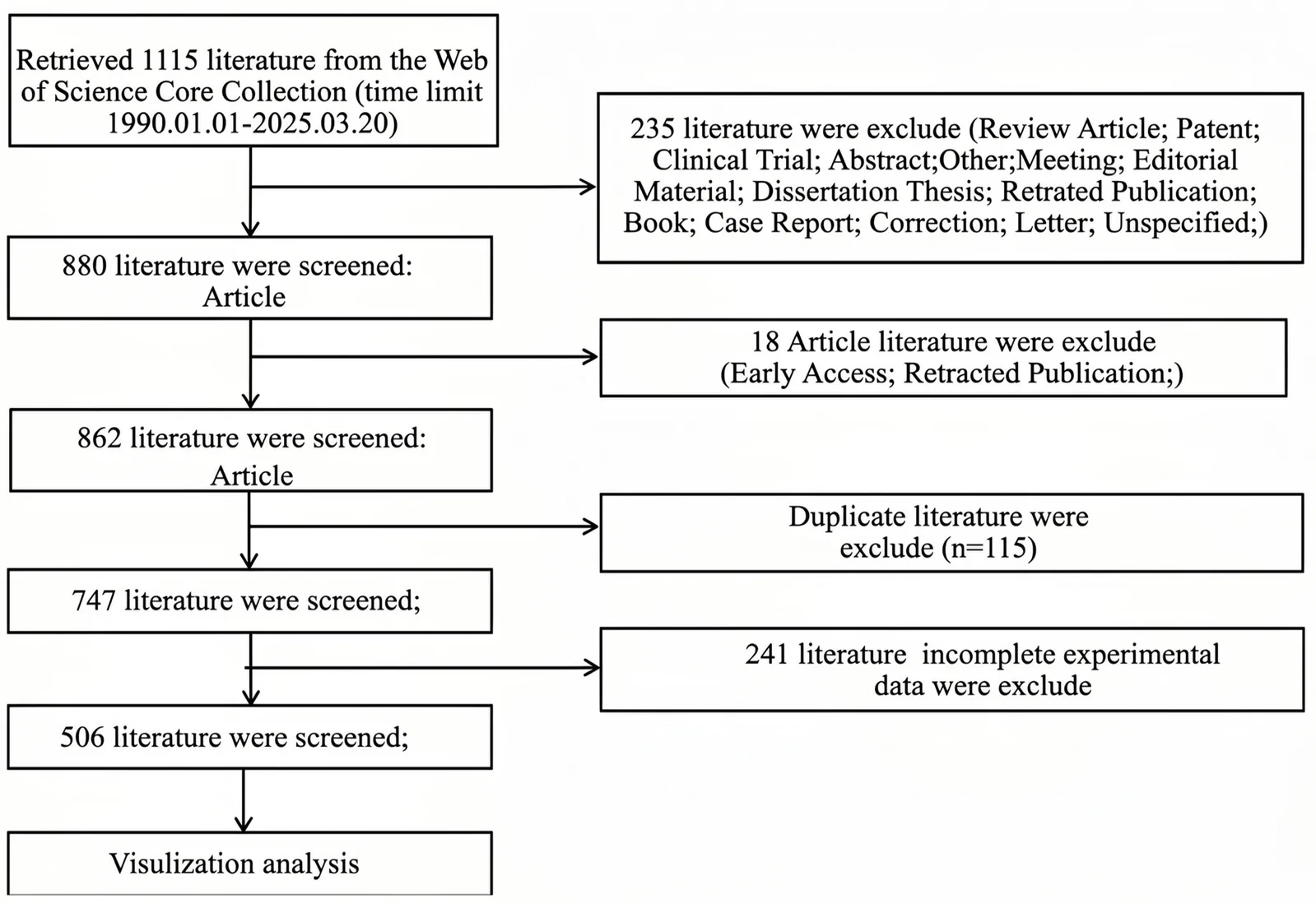
Flow chart of data collection.

The Web of Science Core Collection (WoSCC) database is one of the most systematic, comprehensive, and authoritative databases, featuring over 13,000 globally renowned scholarly publications [14, 18, 19]. It also provides a broad statistical source for bibliometrics software. Therefore, the *WoSCC* database is the most frequently utilized in bibliometric studies. We concentrate on the *WoSCC* to increase the accessibility and representativeness of the data. We use “Topic Search = traditional Chinese medicine OR Herbal OR Medicinal Plants” AND Topic Search = “Ischemic Stroke”, Science Citation Index Expanded (SCI-EXPANDED). Retrieved papers are saved as “plain text” and exported as “full-text citation records”. In this study, the exclusion and inclusion criteria were as follows:

1. The time span of the literature search was from January 1, 1990, to March 20, 2025, covering a period of 35 years.
2. Other kinds of publications were excluded (*e.g*., the Review Article, Patent, Clinical Trial, Abstract, Other, Meeting, Editorial Material, Dissertation Thesis, Retracted Publication, Book, Case Report, Correction, Letter, and Unspecified), and only articles were kept.
3. The Article literature was excluded (Early Access; Retracted Publication; Retracted Publication).
4. Duplicate literature was eliminated.
5. No species limits were set.
6. Only literature that possessed complete experimental data was included (Fig. **1**).

### 2.2 Analysis Method

This paper chooses the version of CiteSpace (6.2.R6W), VOSviewer (version 1.6.20), and Origin2024b as the primary tools to analyze the selected literature comprehensively. Citespace is a citation analysis program based on scientometrics that shows the distribution, trends, and connections of scientific knowledge [20, 21]. Citespace conducted a collaborative network analysis of countries, keyword co-occurrence analysis, and examined the number of publications and keyword frequency. VOSviewer is a distance-dependent tool that enables the visualization of bibliometric networks [22, 23]. The network analysis of the institutions, co-citation analysis of journals and references were calculated using VOSviewer. Origin2025b is used to beautify some result graphics. The number of publications was analyzed using an exponential growth function in Excel [17].

## 3 RESULTS

### 3.1 Overview of Publication Status

We obtained 1115 papers from the WOS, eliminating duplicate literature, review articles, Patents, Clinical Trials, Abstracts, Other Types, Meeting Abstracts, Editorial Materials, Dissertations, Retracted Publications, Early Access Articles, Books, Case Reports, Corrections, Letters, and included only 747 original articles. Secondly, we screened articles with complete experimental data that reported experiments or clinical validations of drug efficacy, including the collection, analysis, and verification of animal or clinical samples. Finally, we obtained 506 original articles. The process of collecting the above articles is shown in Fig. (**1**). Fig. (**2**) displays the annual and cumulative publication counts related to TCM for treating ischemic stroke, showing that they fluctuate slightly overall. From 1 publication in 1992 to 75 publications in 2024, the number of publications increased noticeably, particularly from 2018 (n = 39) to 2021 (n = 54). From January to March 2025, there have been 21 publications in this field. According to this trend, the number of publications in 2025 may be the same as or exceed that of 2024. The annual increase in published articles indicates that TCM treatment is an active research field in ischemic stroke disease and has garnered significant attention from scholars.

### 3.2 Analysis of National Publication Counts

**Fig. (3):**
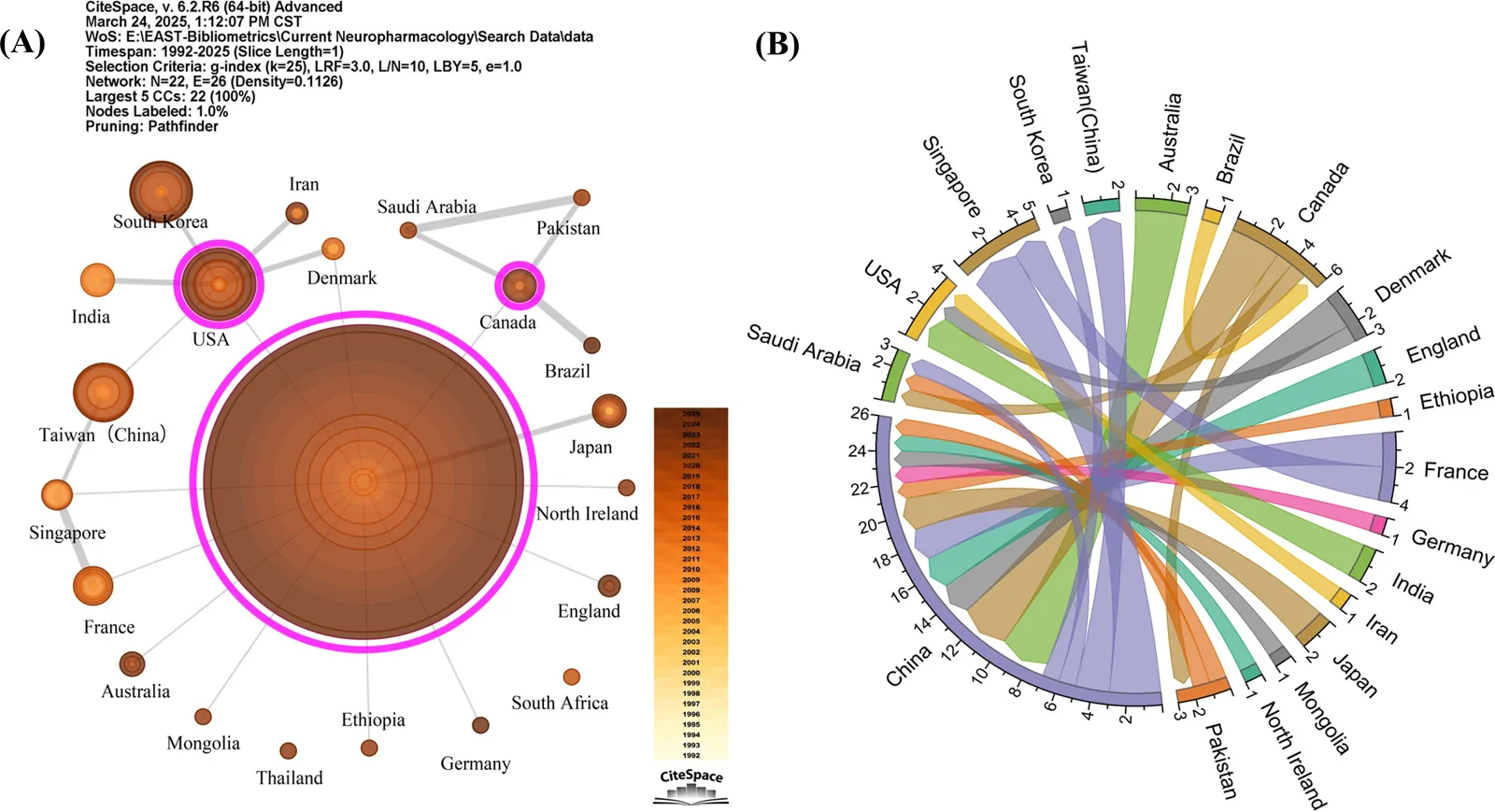
(**A**) The countries of the authors and the collaborative networks among these countries. (**B**) The chord diagram of cooperation frequency between countries.

Fig. (**3**) illustrates the national collaboration network for the research of TCM treatment for ischemic stroke. Twenty different countries have published papers in the relevant topic, according to the CiteSpace analysis of all the data (Fig. **3A**). China claims the highest publication tally (n=418, 97.0%), succeeded by USA (n=25, 5.8%), South Korea (n=21, 4.9%) (Table **S1**). Greater centrality in cooperative networks indicates closer collaboration. The countries with centrality greater than 0.1 are China (1.38), the USA (0.55), and Canada (0.48), meaning that they had the highest percentage of international cooperation with other countries in this research area. As shown in Fig. (**3B**), the chord diagram of cooperation frequency between countries further confirms the above conclusion. The centralities of other countries displayed a lower density, suggesting the relative independence of the research teams and the need for further collaboration.

### 3.3 Contributions of Institutions

Based on citation metrics, Table **S2** lists the top 10 influential organizations. We constructed an institution's collaborative network graph in Fig. (**4A**), presenting the high centrality, critical nodes and explosive nodes of influential organizations. Each node represents a research institution, and the size of the node denotes the quantity of publications. The edges' color reflects the collaboration history, transitioning from orange for recent collaborations to yellow for older ones. We found that the Chinese Academy of Medical Sciences has more publications and higher centrality. Fig. (**4B**) presents a network visualization map that offers insights into institutional collaborations using VOSviewer. China emerged as a key player in this scientific research field with outstanding contributions from institutions, such as the Chinese Academy of Medical Sciences, Beijing University of Chinese Medicine, Capital Medical University, and China Pharmaceutical University. The frequency of co-occurrences between institutions is measured by Total Link Strength (TLS), which indicates the degree of contact and collaboration. The Chinese Academy of Medical Sciences (TLS = 73), Beijing University of Chinese Medicine (TLS = 69), and Capital Medical University (TLS = 65) stand out as the top three institutions with the highest TLS scores.

### 3.4 Analysis of Published Journals

**Fig. (5):**
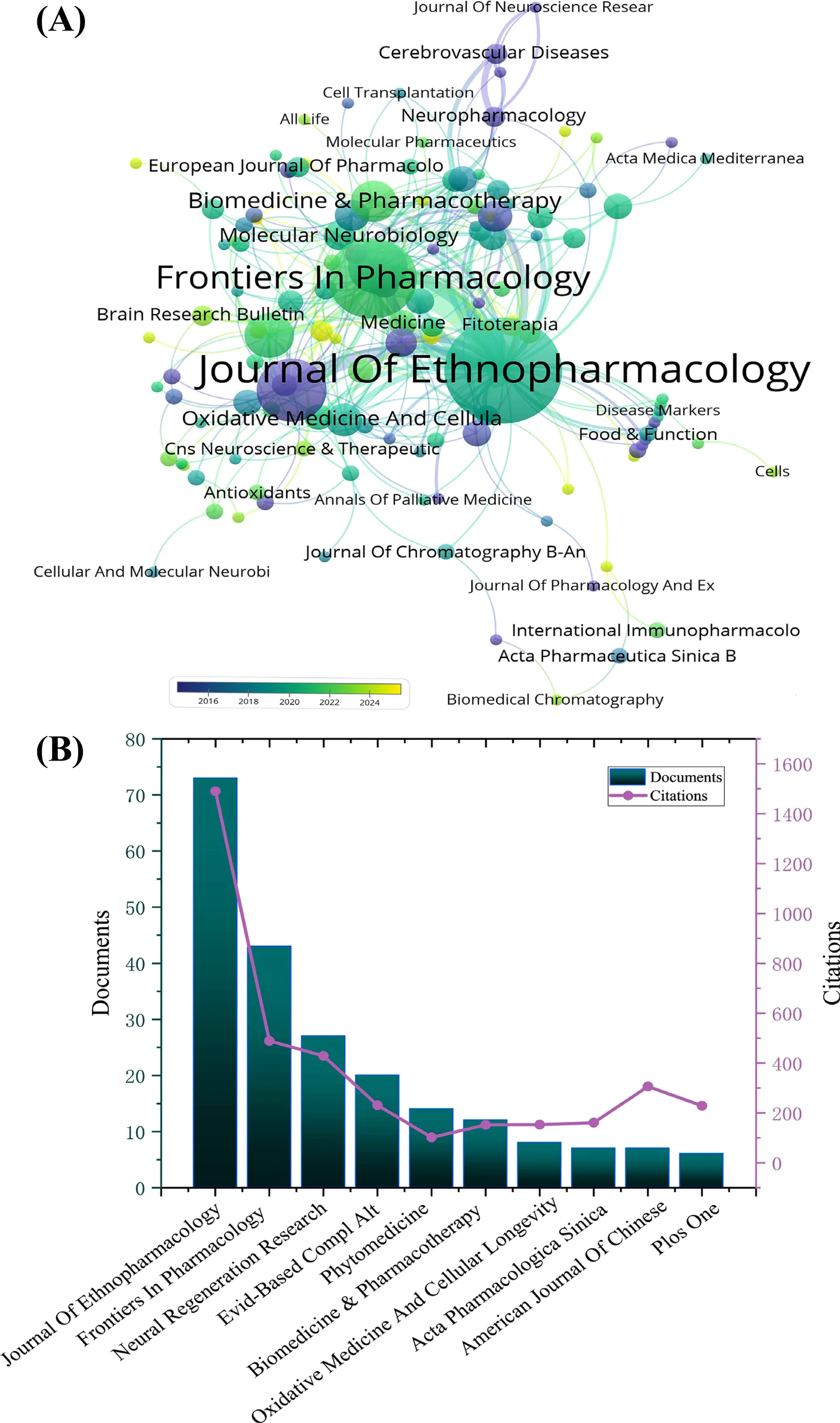
(**A**) The overlay visualization map of published journals from 1990.01.01–2025.03.20. Countries in purple appear earlier than those in yellow; the darker the purple color, the closer it is to the year 1990, and the brighter the yellow color, the closer it is to the year 2025. (**B**) The top 10 journals have the most publications in the field of TCM and ischemic stroke.

We found that 506 articles were published in 177 journals (Fig. **5A**). The time axis of the graph indicates the appearance time and development order of these hotspot journals, progressing from purple to yellow. The top 10 journals with the most articles about TCM in ischemic stroke are listed in Fig. (**5B**). Based on data analysis, we found that the most prolific journals were the Journal of Ethnopharmacology and Frontiersin Pharmacology, which had 73 and 43 documents, respectively. Journal of Ethnopharmacology and Frontiers in Pharmacology might exhibit the greatest influence from the perspective of the number of articles published and the citations.

### 3.5 Emerging Trends and Research Focus based on Keyword Analysis

**Fig. (6):**
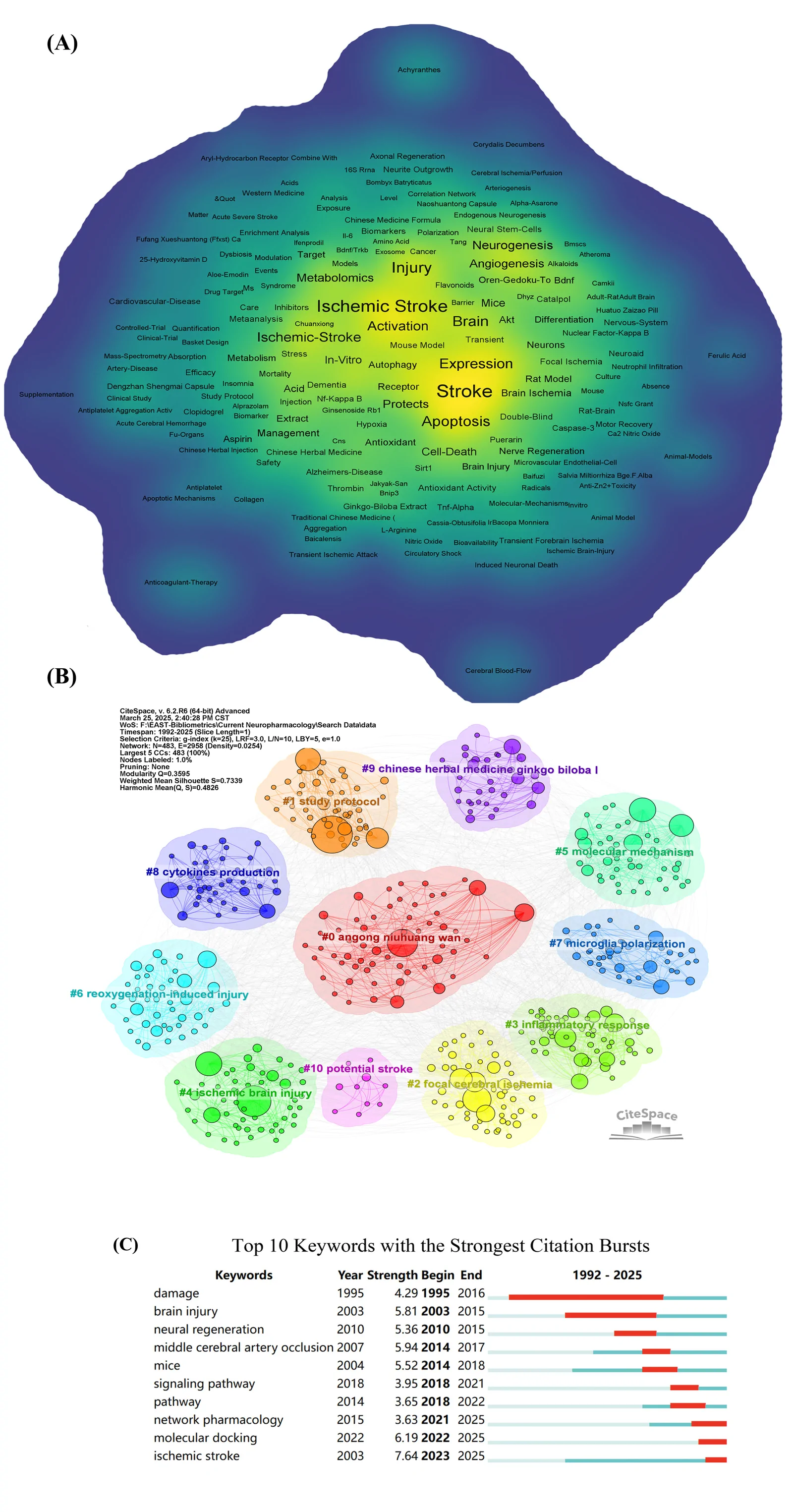
(**A**) VOSviewer visualization for keywords density plot. (**B**) CiteSpace visualization of Keyword Cluster Analysis. (**C**) CiteSpace visualization map of the top 10 keywords with the strongest citation bursts involved in TCM and ischemic stroke.

In the context of bibliometric analysis, keywords usually indicate the primary areas of interest and research hotspots of the author or article, and the development trend in a certain field. After discarding duplicate keywords from 506 articles, a total of 2255 keywords were obtained by VOSviewer (Fig. **6A**), the top 30 Keywords as shown in Table **S3**. Then, we found 11 clusters by cluster analysis of keyword co-occurrence on 2255 keywords (Fig. **6B**). By summarizing the 11 keywords clustering, it could be seen that nerve regeneration was the main research force direction of ischemic stroke, angong niuhuangwan and the constituents of Chinese medicine were the main focus of treating ischemic stroke, network pharmacology was a common method of TCM treatment for Ischemic Stroke.

Fig. (**6C**) presents the top 10 keywords with the strongest citation burst. “Middle cerebral artery occlusion”, “ischemic stroke”, “molecular docking”, and “brain injury” were listed as the four keywords with the highest explosive intensity, and their citation bursts were more than 5.80. It implied that the mouse middle cerebral artery model was the primary model for research, brain injury was the research focus, and molecular docking was a key technology. The trend of keywords showed that “damage” has been a hot topic up until now. In contrast, others have received attention recently and can be considered as frontiers in the field, such as “network pharmacology” (2021-2025), “molecular docking” (2022-2025), and “signaling pathway (2018-2021)”. Frontiers and hotspots share some commonalities.

### 3.6 Emerging Trends and Research Focus based on Terms and Reference Analysis

**Fig. (7):**
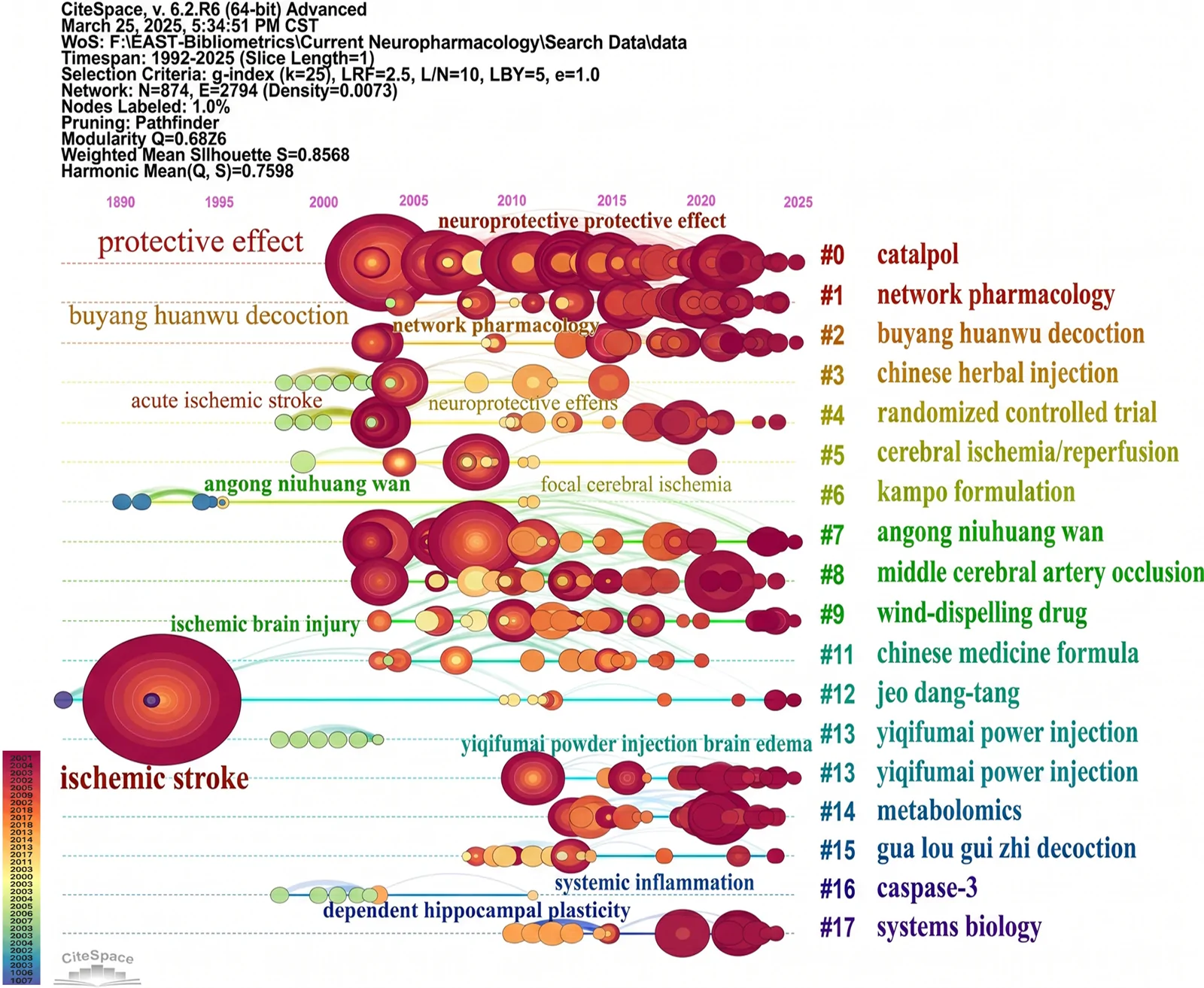
The view of the Cluster and timeline for terms and reference.

We conducted a clustering, statistical, and correlation analysis of terms and co-cited references using CiteSpace. Cluster analysis of title co-occurrence on co-cited references and terms found 18 Clusters, with significant modularity and silhouette scores, suggesting that clusters are highly credible (S = 0.8568, Q = 0.7598) [24]. The 18 clusters are displayed in Table **S4**, including #0 catalpol, #1 network pharmacology, #2 buyang huanwu decoction, #3 chinese herbal injection, #4 randomized controlled trial, #5 cerebral ischemia/reperfusion, #6 kampo formulation, #7 angong niuhuang wan, #8 middle cerebral artery occlusion, #9 neural regeneration, #10 wind-dispelling drug, #11 chinese medicine formula, #12 jeo dang-tang, #13 yiqifumai power injection, #14 metabolomics, #15 gua lou gui zhi decoction, #16 caspase-3, and #17systems biology. In the timeline diagram, terms from the same cluster were arranged in chronological order of their first appearance (Fig. **7**), demonstrating the development of research on TCM for treating ischemic stroke.

According to the cluster structure, catalpol, angong niuhuang wan, buyang huanwu decoction, Jeo-dang-tang, yiqifumai powder injection, and gualou guizhi decoction were the most frequently cited references in the field of TCM for ischemic stroke. Kampo Formulation is an herbal medicine in Japan, which is derived from TCM [25]. Kampo formulations (Oren-gedoku-to (Huanglian Jiedu Tang), Saiko-ka-ryukotsu-borei-to (ChaiHu Jialong Gumu-Litang), and Keishi-bukuryo-gan (Guizhi Fuling wan)) attenuated the post-ischemic brain damage by scavenging free radicals and depressing the generation of lipid peroxidation [26]. As shown in Table **1**, we summarized the components, therapeutic potential, and related mechanisms of the compound TCM and monomeric drugs in the above clustering results. Catalpol is the only monomeric drug cluster and the largest cluster, indicating that catalpol is the most favourable monomeric drug for researching and citing in the study of ischemic stroke, with a high reference value. Next, Buyang Huanwu decoction is the most extensively studied compound TCM, which can promote neurovascular remodeling, immunosuppression, and inhibit pyroptosis and inflammation after stroke [27-29].

## 4 DISCUSSION

Ischemic stroke is a leading cause of disability worldwide, lacking effective drugs to alleviate the illness and suffering of patients at present [7, 39]. After long-term clinical application, TCM has accumulated rich experience and possesses significant potential for future development in stroke treatment [40-42]. Based on the documents retrieved from the *WoSCC*, a total of 506 documents were identified in the research from 1990 to 2025. We summarized and analyzed the published articles in this field of research, which we hope will provide valuable references and insights for the development of novel medicines, as well as for the application of TCM in the prevention and treatment of ischemic stroke.

The trend of published articles provides insight into the pace of advancement and development in a particular field of research [42]. Over the past 35 years, there has been a generally steady increasing trend in the research output related to TCM and ischemic stroke. The literature in this field is not only from Asia and North America but also from Europe and Oceania, suggesting that the use of TCM in ischemic stroke has garnered interest on a global scale. The major contributors in this field are China and the USA. Among them, publications from China were the most and are anticipated to play a significant role in this field. Network cluster analysis showed close cooperation between countries and institutions [43]. The cooperation of the Chinese Academy of Medical Sciences, Beijing University of Chinese Medicine, and Capital Medical University was the closest.

TCM is one of the oldest healing systems and provides a rich basis for modern drug discovery and development [10, 44]. With the rapid growth of TCM, the academic community has spawned many journals related to TCM, including the Journal of Ethnopharmacology, Phytomedicine, and Phytotherapy Research [45-47]. In this study, the Journal of Ethnopharmacology published 73 papers, becoming the highest number of published papers and citations in this field.

At present, the research methods of TCM for treating ischemic stroke mainly include network pharmacology, molecular docking, multi-omics, and molecular biology [48]. This study has repeatedly found through keyword, Terms, and Reference analysis that network pharmacology and molecular docking are high-frequency term in various literature. The combination of network pharmacology and multi-omics is beneficial for predicting the mechanism of drug efficacy. The combination of network pharmacology and molecular biology is used to verify the specific mechanism of action [49, 50]. In addition, Network pharmacology is often combined with molecular docking and analytical chemistry, which helps to analyze the effective ingredients and related targets of TCM [51, 52]. In the era of frequent integration of artificial intelligence multi-omics and algorithms, network pharmacology has ushered in new development opportunities and has helped to bring new breakthroughs in researching diseases and therapeutic drugs.

Ischemic stroke is a complex disease involving multiple aspects such as free radical damage, cellular calcium overload, inflammatory response, and apoptosis [12, 53]. The treatment and repair of post-ischemic stroke is a significant challenge due to the intricate inflammatory milieu and the limited regenerating capacity of neurons [54]. Our research has found that nerve regeneration is currently a hot research direction in treating ischemic stroke with TCM. Multiple compound TCM compounds and monomer compounds have shown advantages in nerve regeneration and neuroprotection, including buyang huanwu decoction [55, 56], angong niuhuang wan [57], naoxinqing tablet [58], houshiheisan [59], pushen capsule [60], and catalpol [61]. saponins [62]. Our findings further substantiate this perspective. In the analysis of keywords, we found that the hot research direction was brain injury, damage, and neural regeneration. This suggests that TCM holds promise in preventing and treating strokes.

Furthermore, this study presents multiple star TCM related to the treatment of ischemic stroke, including buyang huanwu decoction (cluster 2), as well as angong niuhuang wan (cluster 7), yiqifumai powder injection (cluster 13), jeo-dang-tang (cluster 12), and gualou guizhi decoction (cluster 15) from term and reference clustering analysis. Buyang huanwu decoction could improve synaptic plasticity, promote neurovascular remodeling, inhibit pyroptosis, and alleviate stroke-induced immunosuppression [27-29, 63]. The exerts therapeutic effects of buyang huanwu decoction on ischemic stroke through the cAMP/PKA/CREB pathway, astrocyte and microglia polarization, and cell apoptosis [29, 64, 65]. Angong niuhuang wan reduced the BBB damage, inhibited ferroptosis, reduced hemorrhagic transformation, and enhanced neuroprotective effects in ischemic stroke [30, 66, 67]. Its mechanism is mainly related to activating the PPARgamma/AKT/GPX4 pathway [30]. Similarly, based on their neuroprotective effects, gualou guizhi decoction, Jeo dang-tang, and yiqifumai powder injection are also widely used for the treatment of ischemic stroke [31, 32, 68-70]. Gualou guizhi decoction exhibits neuroprotective effects on post-stroke spasticity by modulating glutamate levels and AMPA receptor expression, and it also attenuates post-stroke spasticity through the modulation of GABAB receptors [69, 71, 72]. Yiqifumai powder injection ameliorates blood-brain barrier dysfunction and brain edema after stroke despite an unclear mechanism [68]. In addition, this study presents a catalpol cluster related to the treatment of ischemic stroke from term and reference clustering analysis. Catalpol, an iridoid glucoside isolated from Rehmannia glutinosa, exhibits anti-inflammatory, antioxidant, and neurogenic properties, promoting neurogenesis and angiogenesis, and is used in the treatment of ischemic stroke [37, 61, 73, 74]. Catalpol promotes neurogenesis and angiogenesis *via* the SDF-1α/CXCR4 pathway, thereby enhancing VEGF-PI3K/AKT signaling [37, 61].

In the study of the compound mentioned above TCM researchers identified multiple monomeric compounds with therapeutic potential for ischemic stroke, such as perulic acid, propyl gallate, calycosin, and pormononetin [75, 76]. However, the therapeutic potential and related mechanisms of these monomeric compounds need further exploration and research. Thus, further exploration of the pharmacological effects and mechanisms of monomeric compounds in TCM will likely be a new hotspot in future ischemic stroke treatment.

In addition, this study also has some limitations. First, TCM publications treating ischemic stroke-related conditions are being continually published and cited, and the bibliometric results might change since we completed the analyses. In order to minimize the impact of this drawback, we have extended the time frame for literature collection to the date of manuscript revision. Second, it could be less comprehensive to use keywords to infer content. Although keywords can indicate a topic's relevance, they are unable to precisely capture the ideas included in articles, which could lead to errors in bibliometric study reporting. However, our subsequent terms and reference analysis can partially rectify this issue. Finally, the self-reference bias needs to be carefully considered and assessed. In the future, we will further expand relevant data more accurately and obtain more compelling research results with the update of literature management technology and retrieval methods.

## CONCLUSION

This paper, to our knowledge, reviews the current literature on TCM in ischemic stroke and provides the first knowledge structure and emerging research trends in the field using bibliometrics. Our result demonstrated that the field of TCM and ischemic stroke is in a state of sustained development. In addition, the active ingredients or monomers of TCM are forefronts that deserve constant attention.

## Figures and Tables

**Fig. (2):**
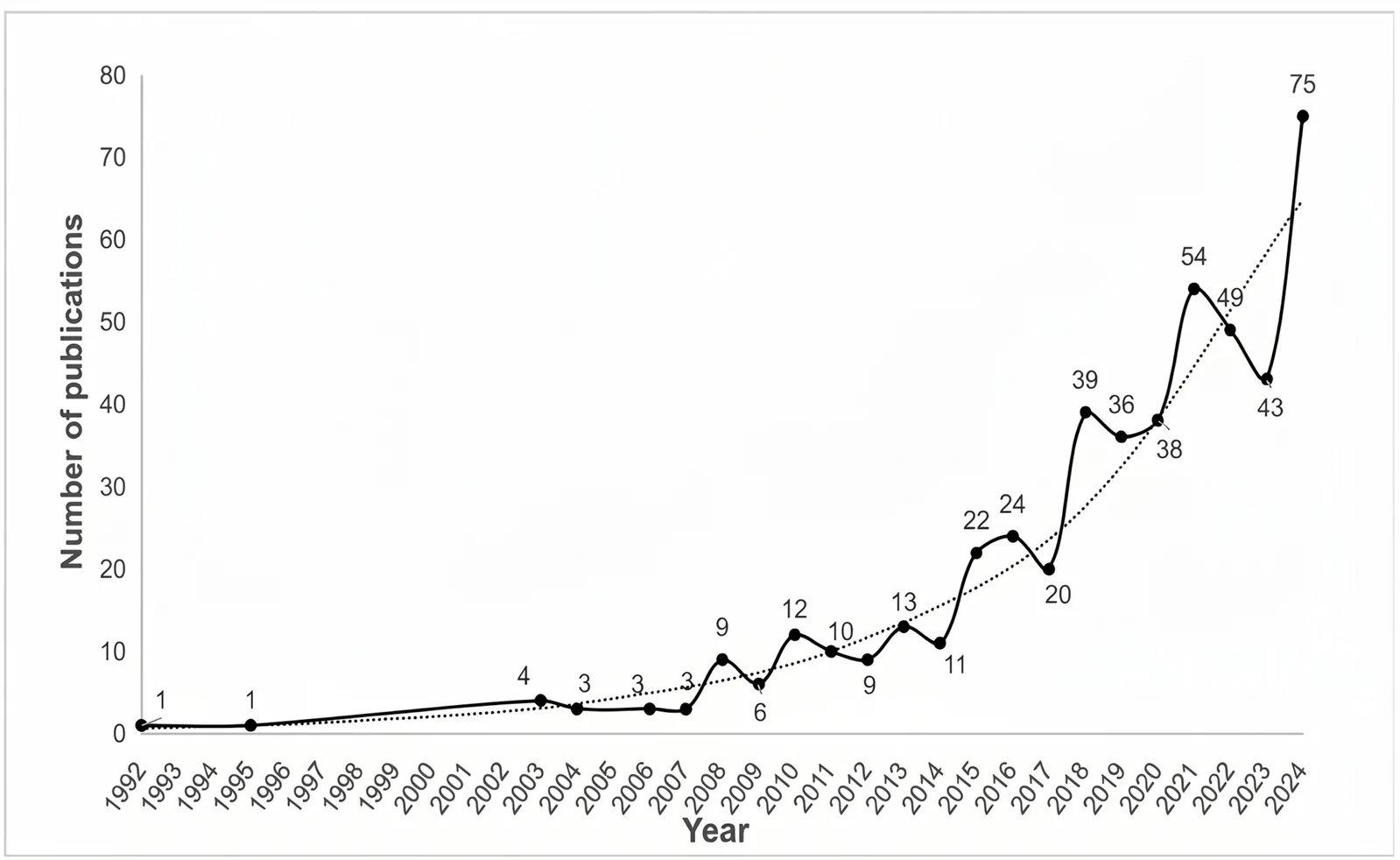
Annual publication volume of Chinese medicine treatment for Ischemic Stroke from 1992 to 2024.

**Fig. (4):**
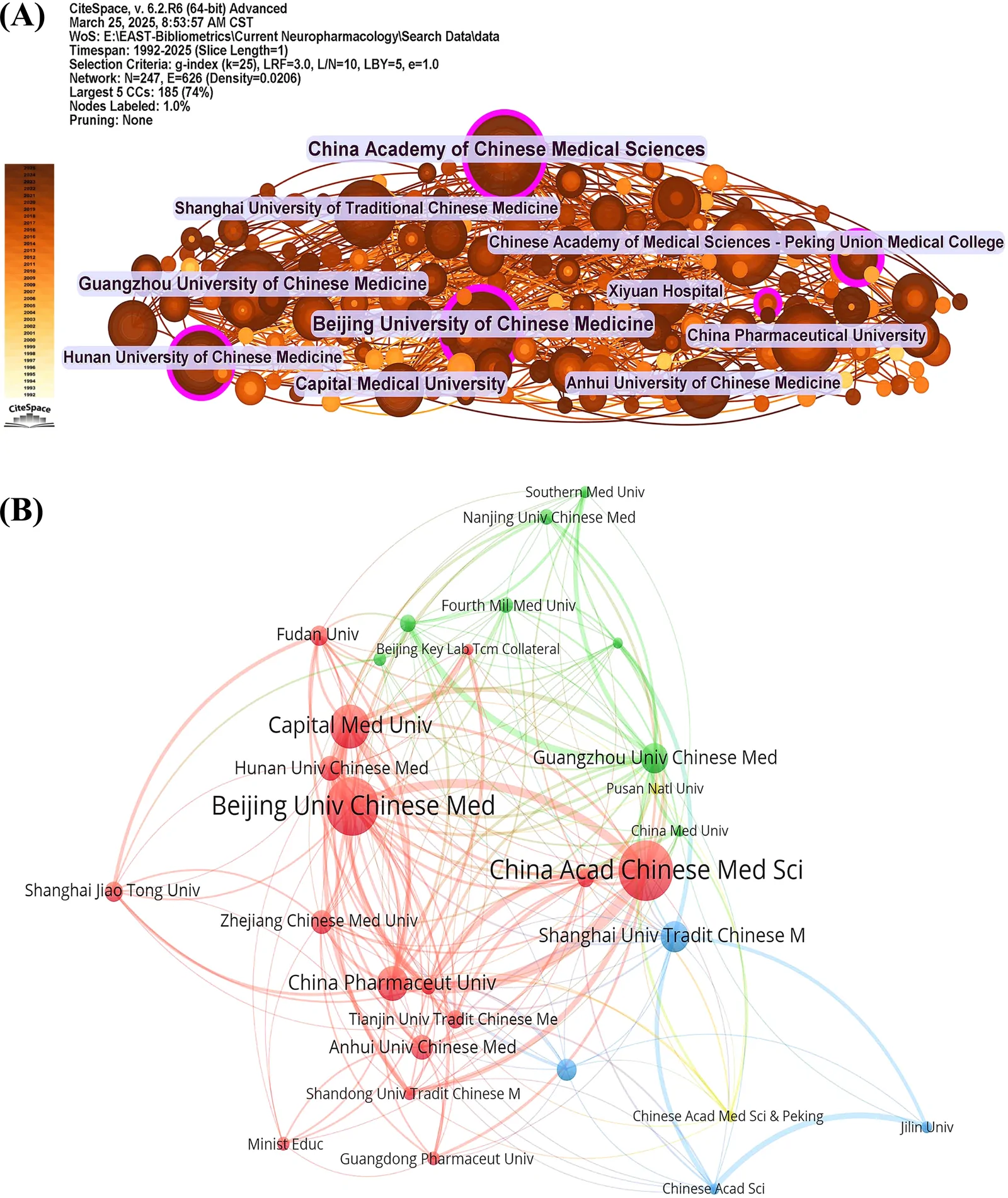
(**A**) The institution cooperation network. (**B**) Citation of selected 30 organizations.

**Table 1:** The ingredients and mechanisms related to TCM for treating ischemic stroke.

**Dosage Form**	**Drug Name**	**Ingredient**	**Therapeutic Potential**	**Related Mechanisms**	**References**
Compound TCM (or Kampo Formulation, Japan [26])	Buyang Huanwu Decoction	-	Promoting neurovascular remodeling, inducing immunosuppression, inhibiting pyroptosis and inflammation	The cAMP/PKA/CREB pathway, AIM2 inflammasome, and the PINK1/ Parkin mitophagy pathway.	[27-29]
	Angong Niuhuang Wan	-	Neuroprotective, reducing hemorrhagic transformation and mortality	Activating PPARγ/AKT/ GPX4 pathway	[30]
	Yiqifumai powder injection	-	Ameliorating blood-brain barrier dysfunction and brain edema	ROCK1 and NF-κB signaling pathways	[31]
	Gua Lou Gui Zhi decoction	-	Neuroprotection attenuates post-stroke spasticity	The TLR4/NF-κB pathway	[32]
	Jeo Dang-Tang	-	Being used for various cerebrovascular diseases	Increasing the LPS or PHA-induced TGF-β1 production	[33]
	Huanglian Jiedutang	Total alkaloids, flavonoids, and iridoids	Protective autophagy, neuroprotection	MAPK-mTOR signaling pathway.	[34]
	Chaihu Jialong Gumu-Litang	-	Treating post-stroke depression	-	[35]
	Guizhi Fuling Wan	-	Treating cold sensation and numbness after a stroke	Inhibit the free radical production	[36]
monOmer	Catalpol	-	Promoting neurogenesis and angiogenesis	The SDF-1α/CXCR4 pathway, the VEGF-A/KDR Pathway	[37, 38]

## Data Availability

Not applicable.

## LIST OF ABBREVIATIONS

TCM: Traditional Chinese Medicine
TLS: Total Link Strength
WoSCC: Web of Science Core Collection
