# Supplementary material for: Research Hotspots and Frontiers of Ischemic Stroke and Traditional Chinese Medicine: A Knowledge Mapping and Text Mining Analysis
Source: Curr Neuropharmacol. 2025 Jul 18;24(6):838–50. doi: 10.2174/011570159X378811250702095624 (PMC13519839; doi:10.2174/011570159X378811250702095624)
Supplement: Supplementary file 1 [file CN-24-6-838_SD1.pdf]

Supplementary Material

Research Hotspots and Frontiers of Ischemic Stroke and Traditional Chinese Medicine: A Knowledge Mapping and Text Mining Analysis

Jian Xie[1], Teng Li[2] and Yuan Zhang[3,*]

[1]Department of Neurology in Affiliated ZhongDa Hospital and Jiangsu Provincial Medical Key Discipline, School of Medicine, Institution of Neuropsychiatry, Key Laboratory of Developmental Genes and Human Disease, Southeast University, Nanjing, Jiangsu, 210009, China; [2]Department of Laboratory Medicine, Shanghai East Hospital, School of Medicine, Tongji University, Shanghai, 200120, China; [3]Department of Pharmacy, Shanghai East Hospital, Tongji University School of Medicine, Shanghai, 200120, China

Table S1. Top 10 countries with research of TCM treatment for ischemic stroke from 1990.01.01 to 2025.03.20.

Rank	Article Counts	Percentage 100%	Centrality	Countries
1	418	97.0	1.38	China
2	25	5.8	0.55	USA
3	21	4.9	0	South Korea
4	19	4.4	0.02	Taiwan (China)
5	7	1.6	0	France
6	6	1.4	0	Japan
7	6	1.4	0	India
8	5	1.2	0.07	Singapore
9	4	0.9	0.48	Canada
10	3	0.7	0	Australia
11	2	0.5	0	Denmark
12	2	0.5	0	Iran
13	2	0.5	0	England
14	1	0.2	0	South Africa
15	1	0.2	0	Pakistan
16	1	0.2	0	Ethiopia
17	1	0.2	0	North Ireland
18	1	0.2	0	Brazil
19	1	0.2	0	Thailand
20	1	0.2	0	Germany

1875-6190/26 $65.00+.00 © 2026 Bentham Science Publishers

Table S2. The top 10 institutions with the most influential institutions in the field of TCM and Ischemic Stroke.

Rank	Name	Country	Article Counts	Total Link Strength	Centrality
1	Chinese Academy of Medical Sciences	China	38	73	0.16
2	Beijing University of Chinese Medicine	China	37	69	0.22
3	Capital Medical University	China	28	65	0.10
4	China Pharmaceutical University	China	22	61	0.04
5	Shanghai University of Traditional Chinese Medicine	China	20	34	0.05
6	Anhui University Chinese Medicine	China	16	25	0.04
7	Zhejiang Chinese Medicine University	China	15	28	0.01
8	Fudan University	China	13	32	0.05
9	Chengdu University Traditional Chinese Medicine	China	12	31	0.01
10	University Hong Kong	China	11	29	0.03

Total link strength is computed using VOSviewer, whereas centrality is calculated using Citespace.

Table S3. Top 30 Keywords with research of TCM treatment for ischemic stroke from 1990.01.01 to 2025.03.20.

Rank	Keywords	Occurrences	Total Link Strength
1	Stroke	149	563
2	Ischemic stroke	123	466
3	Injury	83	365
4	Oxidative stress	70	319
5	Traditional chinese medicine	70	246
6	Brain	67	286
7	Apoptosis	66	326
8	Neuroprotection	65	322
9	Expression	60	288
10	Ischemic-stroke	56	175
11	Cerebral-ischemia	51	207
12	Inflammation	51	237
13	Mechanisms	51	232
14	Activation	48	240
15	Cerebral ischemia	45	199
16	Artery occlusion	44	202
17	Cells	37	192
18	Rats	37	173
19	Model	36	163
20	Inhibition	35	162
21	Network pharmacology	35	144

Rank	Keywords	Occurrences	Total Link Strength
22	Neurogenesis	34	162
23	Acute ischemic-stroke	32	120
24	Damage	32	157
25	Angiogenesis	30	130
26	Focal cerebral-ischemia	30	144
27	Protects	30	151
28	Reperfusion injury	30	144
29	Metabolomics	28	103
30	Ischemia	51	237

Table S4. Terms cluster analysis.

Cluster ID	Size	Silhouette	Average Year	Label
0	99	0.690	2015	Catalpol
1	82	0.764	2017	Network pharmacology
2	62	0.699	2018	Buyang huanwu decoction
3	62	0.983	2003	Chinese herbal injection
4	61	0.864	2009	Randomized controlled trial
5	44	0.934	2008	Cerebral ischemia/reperfusion
6	44	0.978	1998	Kampo formulation
7	41	0.931	2016	Angong niuhuang wan
8	40	0.829	2014	Middle cerebral artery occlusion
9	40	0.835	2014	Neural regeneration
10	33	0.785	2011	Wind-dispelling drug
11	32	0.901	2001	Chinese medicine formula
12	32	1	2002	Jeo dang-tang
13	29	0.836	2021	Yiqifumai powder injection
14	28	0.889	2019	Metabolomics
15	25	0.900	2013	Gua lou gui zhi decoction
16	23	0.983	2002	Caspase-3
17	21	0.925	2017	Systems biology
